# Lichen Planus Pemphigoides: From Lichenoid Inflammation to Autoantibody-Mediated Blistering

**DOI:** 10.3389/fimmu.2019.01389

**Published:** 2019-07-02

**Authors:** Franziska Hübner, Ewan A. Langan, Andreas Recke

**Affiliations:** ^1^Department of Dermatology, University of Lübeck, Lübeck, Germany; ^2^Dermatological Sciences, University of Manchester, Manchester, United Kingdom; ^3^Lübeck Institute of Dermatological Research, University of Lübeck, Lübeck, Germany

**Keywords:** Lichen planus pemphigoides, autoantibodies, Bullous Pemphigoid, Lichen Planus, BP180, BP230

## Abstract

Lichen planus pemphigoides (LPP) is a very rare autoimmune sub-epidermal blistering disease associated with lichenoid skin changes. Initially thought to be a mere variant of more common inflammatory dermatoses, particularly Bullous Pemphigoid (BP) or Lichen Planus (LP), a growing body of evidence suggests that it is a disease entity in its own right. In common with a range of autoimmune blistering diseases, including BP, pemphigoid gestationis (PG), mucous membrane pemphigoid (MMP) and linear IgA dermatosis (LAD), a key feature of the disease is the development of autoantibodies against type XVII collagen (COL17). However, accurately establishing the diagnosis is dependent on a careful correlation between the clinical, histological and immunological features of the disease. Therefore, we present an up to date summary of the epidemiology and etiopathogenesis of LPP, before illustrating the predisposing and precipitating factors implicated in the development of the disease. In addition to a selective literature search, we compare reports of potential drug-induced cases of LPP with pharmacovigilance data available via OpenVigil. We subsequently outline the cardinal clinical features, important differential diagnoses and current treatment options. We conclude by demonstrating that an improved understanding of LPP may not only lead to the development of novel treatment strategies for the disease itself, but may also shed new light on the pathophysiology of more common and treatment-refractory autoimmune blistering diseases.

## Introduction

First described by Kaposi over a century ago (1), Lichen planus pemphigoides (LPP, syn. Lichen ruber pemphigoides) is commonly considered to be a variant of Lichen Planus (LP), characterized and complicated by the formation of tense blisters and bullae.

In addition to the clinical findings, including lichenoid plaques and tense blisters, the gold standard for the diagnosis of LPP is the demonstration of autoantibody deposition along the dermal-epidermal junctional zone in perilesional skin biopsies; first reported by Stingl and Holubar in 1975 (2). Almost two decades later, Tamada et al. established that the autoantigen in LPP is a 180 kDa protein expressed in the hemidesmosomes of the dermal-epidermal junction (3). Interestingly, the same autoantigen is responsible for the development of Bullous Pemphigoid (BP), namely type XVII collagen (COL17) (4).

## Definition

Lichen planus pemphigoides can be best defined as an autoimmune dermatosis, the hallmarks of which are lichenoid and bullous skin lesions, which develop in the context of autoantibodies targeting type XVII collagen COL17.

Clinically, the diagnosis relies on carefully distinguishing the disease phenotype from that seen in bullous LP. Given the potential clinical, histological and immunological overlap between LPP, bullous LP and BP, the clinician must also determine which disease is present, although they may occasionally occur simultaneous, which may further complicate reaching the correct diagnosis(ses). Several clinical features can support reaching the correct diagnosis. For example, bullous LP classically describes the formation of blisters on pre-existing lichenoid plaques. In contrast to bullous LP, the blisters of LPP are typically located outside of LP lesions. However, several cases of LPP with blistering restricted to the lichenoid plaques have been reported, casting doubt on the utility of blister localization to clinically differentiate between LPP and Bullous LP with any degree of certainty (5, 6). The blisters in Bullous Pemphigoid tend to occur on urticated plaques and may evolve into erosions and crusts.

Moreover, there are other subtle differences in the clinical presentation of BP and LPP. For example, the typical age of onset of LPP is significantly younger than that in BP (7, 8). Furthermore, LPP lesions are predominantly found on the extremities, whereas in BP they are more often generalized (8) and associated with pruritus. The clinical course of LPP is usually less protracted and generally milder than that in BP.

Ultimately, the detection of autoantibodies to the dermal-epidermal junction is central to supporting and securing the diagnosis. In contrast to LPP, the skin changes seen in bullous LP develop in the absence of autoantibodies against structural proteins of the skin, especially COL17 (9). In terms of pathogenesis, given that in the majority of cases of LPP the development of lichenoid skin lesions precedes the formation of blisters, it has been hypothesized that lichenoid inflammation itself may actually promote the development of an autoimmune response, targeting proteins of the epidermal basement membrane (10–14).

In fact, Kromminga et al. identified subtle differences in the epitope specificity of autoantibodies in the sera of patients with LPP, BP, and mucous membrane pemphigoid (MMP) (15), using recombinant fragments of the NC16A subdomain of COL17. The sera of 12 patients with BP, 6 with gestational pemphigoid (PG), 10 with MMP and 4 with LPP were examined using nine overlapping dihydrofolate reductase-fused subfragments with a size of 13–18 amino acids of the NC16A (amino acids E_490_-L_565_ of COL17, Uniprot entry Q9UMD9) to evaluate the epitope binding pattern by immunoblotting (15). Here, most BP and PG patient sera bound to fragments representing amino acids E_490_-G_532;_ MMP patient sera preferentially bound to fragments E_490_-R_507_ and D_514_-L_565_; while LPP sera generally lacked binding to E_490_-R_507_ but showed reactivity with fragments comprising D_514_-L_565_. Kromminga et al. did not perform any statistical evaluation as to whether these differences are significant. However, on the basis of the published data it was possible to carry out a statistical analysis^1^. We found that the differences between BP and LPP (*p* = 0.0032), MMP and LPP (*p* = 1.07 × 10^−13^) and LPP and PG (*p* = 3.08 × 10^−43^) were actually highly significant. In addition, BP and PG (*p* = 3.82 × 10^−13^) and MMP and PG (*p* = 1.95 × 10^−24^) had significantly different binding patterns, while MMP and BP did not.

## Epidemiology

The exact prevalence of LPP is unknown. Only 4 cases of LPP were identified in a cohort of 68 patients with blistering diseases from Kuwait; equivalent to an incidence of 0.3/1,000,000 inhabitants (16). A study from India reported 3 patients with LPP in a series of 268 cases with autoimmune blistering dermatoses (17). In contrast, epidemiological studies in patients with blistering dermatoses, based in France, Germany, Greece, Serbia, and Singapore, with patient numbers ranging from 41 to 1,161, did not identify any cases of LPP (18–23). Based on ICD10 classification data from health insurance providers in Germany, the reported prevalence of L12.8 (other pemphigoid diseases) was 4.7 per million patients and 259 per million patients for BP (L12.0) (7). Unfortunately, the LPP ICD10 code L43.1, was not specifically evaluated. However, the epidemiological data analysis based upon ICD10 codes is complicated by the fact that the ICD10 code L43.1 is shared between LPP and bullous LP. Nevertheless, based on the available data the prevalence may be estimated at about 1 per 1,000,000 patients.

The sex ratio (male/female) is described to be roughly 0.8/1 in adults and 3.3/1 in children and adolescents (8), failing to support a specific predilection according to sex. The mean age of onset is approximately 46 years (range between 4 and 85), which is well below the typical age of onset of BP (7). Interestingly, it is not exceptionally rare for LPP to affect children and adolescents. Indeed, in a case report collection with 78 patients, 13 (~16%) were children or adolescents (8).

## Etiopathogenesis

LPP is characterized by autoantibodies against type XVII collagen (COL17, BPAG2), a structural protein that resides in hemidesmosomes at the dermal-epidermal junction (4, 24, 25). Similarly to BP, autoantibodies in LPP may also bind to the 230 kDa BPAG1 (3). In most cases, the COL17-specific autoantibodies in LPP react with the membrane-proximal NC16A subdomain (amino acid residues 490–565 of UniProt entry Q9UMD9) (4, 24). In addition, the C-terminal portion of COL17 and desmoglein 1 have been identified as epitopes and antigens, respectively, in LPP (26). Other autoantibodies against unidentified antigens with a molecular weight of 130 kDa (27) and 200 kDa (28) have also been described. The reported variability in autoantigen specificity may result in clinical variants of LPP which appear similar to BP, with autoantibodies against NC16A (24, 29), and MMP with mucosal lesions and autoantibodies against the C-terminal portion of COL17 (26, 30).

In fact, COL17 is a common autoantigen in a variety of autoimmune blistering dermatoses (31, 32), including LPP, BP, linear IgA dermatosis (33, 34), PG, MMP and paraneoplastic pemphigus (35). Autoantibodies against COL17 have been demonstrated to induce inflammation and blistering due to the effector functions of the Fc portion (36–38). Moreover, a deposition of complement factor C3 at the dermal-epidermal junction found in skin biopsies of LPP indicates an involvement of complement in the pathogenesis. In case of BP and epidermolysis bullosa acquisita, a similar subepidermal blistering disease, the activation of the complement system has been described as a crucial event in the pathogenesis (39, 40).

However, a growing evidence suggests that both complement-dependent and complement-independent mechanisms may both be relevant and effective in subepidermal blistering dermatoses (41–44). The amount of complement-activating IgG1 and non-activating IgG4 autoantibodies (45) is variable between patients. Cases with only IgG4 autoantibodies and without any complement deposition at the derma-epidermal junction exist, suggesting complement-independent mechanisms in blister formation (43). Binding of leporine autoantibodies to type XVII collagen was demonstrated to induce skin fragility, both in a complement-dependent and independent manner, in a murine model for bullous pemphigoid (44). This was confirmed in a similar mouse model, additionally indicating a disease mitigating effect of complement receptor C5aR2 (42).

Although not specifically demonstrated for all diseases, the pathogenic mechanisms ultimately resulting in subepidermal cleavage and macroscopic may be similar and/or shared between distinct autoimmune blistering dermatoses.

The development of autoantibodies against COL17 in LP appears to be a primary event in the development of LPP. This is supported by the fact that blistering almost exclusively follows the appearance of the typical lichenoid skin lesions (8).

In more than 40% of cases with vulvar LP, the NC16A domain of COL17 has been demonstrated to be a target for circulating T cells with rapid effector function (10). The authors used an ELISpot assay that detected IFNγ-producing autoreactive T cells, i.e., the only determined Th1 responses. The T-cell responses in bullous pemphigoid have been investigated with more detail: it appears that most patients have a Th2 or mixed Th1/Th2 response to the complete extracellular portion of BP180 (46). The situation of T cell responses in LPP is unknown. One may speculate that the Th1 response in LP could protect from conversion to a blistering disease, and that a dysregulated Th2 response to NC16A might be necessary for the development of LPP.

In addition to T cell responses, several authors have reported the presence of circulating autoantibodies against COL17 in patients with LP, especially oral or genital LP (10–14). A clinical course similar to LPP was observed in LP-type chronic graft-vs.-host disease, where the patient developed autoantibodies against COL17 and mucous membrane pemphigoid (47). In summary, the development of autoantibodies against COL17 in LPP appears to be linked to the T cell-mediated lichenoid inflammation.

## Clinical Features and Establishment of Diagnosis

Diagnosis is based on careful correlation of the clinical, histopathological and immunopathological features.

In LPP, two discrete primary skin lesions occur: lichenoid papules/plaques and tense blisters. Cases of LPP exclusively restricted to mucous membranes have also been reported (48, 49). The nail apparatus may also be affected by LPP, resulting in nail atrophy or even loss of the nail plate (50). This suggests that LPP is in fact a very heterogeneous disease, whose clinical symptoms and immunologic markers can mimic both BP and mucous membrane pemphigoid (49).

**Figure 1:**
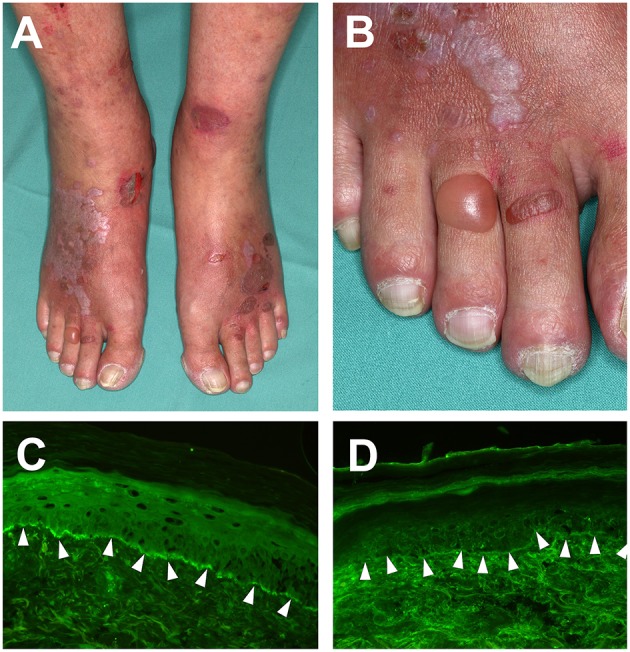
Clinical features of Lichen planus pemphigoides. **(A)** Violaceous plaques with polygonal configuration affecting the dorsal aspects of the feet, with tense blisters and erosions on non-lesional skin. **(B)** Close-up view of the right foot. **(C)** detection of complement factor C3 deposition and, **(D)** IgG deposition at the dermal-epidermal junction in a punch biopsy from perilesional skin, using direct immunofluorescence microscopy (magnification 200×).

The lichenoid eruption consists of pruritic violaceous polygonal papules and plaques with a shiny surface (Figures 1A,B). On the mucosa, patterned white streaks may be found, most prominently on the buccal and the outer genital mucosa (48, 51). Blisters and erosions typically appear after the development of the lichenoid skin changes and classically on previously unaffected skin. Histopathology of a bullous lesion shows the typical feature of BP (2, 31): subepidermal separation with multiple eosinophils in the blister fluid and an eosinophilic infiltrate, whereas histopathology of a lichenoid lesion shows the typical features of lichen planus with focal hyperkeratosis, hypergranulosis, subepidermal band-like lymphocytic infiltrate, and Interface dermatitis with vacuolar change at the dermal-epidermal junction and apoptotic keratinocytes (so called Civatte bodies) (2, 51, 52).

IgG and complement factor C3 deposition at the dermal-epidermal junction zone can be detected using direct immunofluorescence of perilesional biopsies (2) (Figures 1C,D). In contrast, direct immunofluorescence studies of lichenoid lesions may show typical features of LP: irregular band of fibrinogen at the dermal-epidermal junction and colloid bodies in the papillary dermis (53). Circulating autoantibodies in patient sera that bind to the NC16A or C-terminal domains of COL17 or desmogleins may be detected using routine methods including indirect immunofluorescence on monkey esophagus or human neonatal foreskin (54), ELISA and immunoblotting of human keratinocyte extracts (32, 55). In addition to this, circulating autoantibodies will bind to the roof of the artificial blister in indirect immunofluorescence on 1M NaCl-salt split skin (56).

The gold standard for the diagnosis of LPP is the combination of the typical clinical features and the demonstration of autoantibodies binding to the dermal-epidermal junction.

## Differential Diagnosis

In addition to BP and bullous LP, several other dermatoses form the differential diagnosis. For example, erythema multiforme has morpho- and histo-logical features that resemble LPP, most notably a lichenoid or interface dermatitis and blistering (57).

Atypical subacute cutaneous lupus erythematosus may present with similar clinical features as LPP (57, 58), underscoring the importance of a complete immunologic work-up to demonstrate autoantibodies against structural proteins of the skin, and especially the NC16A domain of BP180. Furthermore, in case of atypical clinical presentation, antinuclear antibody and dsDNA antibody testing is recommended.

Paraneoplastic pemphigus may present with similar skin changes and immunological features are those in LPP (59, 60), including autoantibodies against BP180. However, paraneoplastic pemphigus serum autoantibodies typically cause an intercellular binding pattern in the epidermis and can bind to rat bladder epithelia and to envoplakin and periplakin (61).

During the course of disease, LPP may even evolve into other types of autoimmune blistering skin diseases, such as pemphigoid nodularis (62), also known as non-bullous pemphigoid.

## Drugs and Conditions Associated with LPP

Several reports describe an association between the development of LPP and medication and/or and pre-existing medical conditions.

### LPP and Drugs

Relying solely on observational evidence, several drugs have been associated with the development of LPP (Table 1). A causal association is at least conceivable when the LPP developed shortly after the intake of new medication and fully resolved after cessation of the suspected drug-trigger. Perhaps the strongest evidence for drug-induced LPP is in the context angiotensin-converting enzyme (ACE) inhibitor use. There are four reports of an association of LPP with different angiotensin-converting enzyme (ACE) inhibitors (64, 67, 68, 74). Nevertheless, it should be acknowledged that in the majority of published reports of medication-induced LPP, drug re-challenge was either not performed or not associated with disease recurrence.

**Table 1:** Drugs and conditions that were reported to be associated with LPP.

**Drug**	**Reference^c^**	**Pharmacovigilance data** **disproportionality analysis**^a,^ ^b^			
		**Lichen planus**		**Pemphigoid**	
		**Chi squared (Yates)**	**Reporting odds ratio (95%-CI)**	**Chi squared (yates)**	**Reporting odds ratio (95%-CI)**
Cinnarizine	(63)	n.r.	n.r.	n.r.	n.r.
**Enalapril**	(64)	7.93^*^	**3.1 (1.48–6.53)**	35.93^*^	**3.79 (2.41–5.95)**
Narrowband UVB	(27)	*n.a*.	*n.a*.	*n.a*.	*n.a*.
Chinese herbs	(65)	*n.a*.	*n.a*.	*n.a*.	*n.a*.
“weight reduction drug”	(66)	*n.a*.	*n.a*.	*n.a*.	*n.a*.
**Captopril**	(67, 68)	17.93^*^	**6.48 (2.69–15.62)**	0.03	0.58 (0.08–4.11)
**Simvastatin**	(69)	11.09^*^	**1.79 (1.28–2.51)**	51.79^*^	**2.13 (1.73–2.62)**
**Pembrolizumab**	(70)	88.22^*^	**10.1 (5.71–17.83)**	427.24^*^	**14.06 (10.15–19.46)**
PUVA	(71)	*n.a*.	*n.a*.	*n.a*.	*n.a*.
Varicella Chickenpox	(72, 73)	*n.a*.	*n.a*.	*n.a*.	*n.a*.
**Ramipril**	(74)	43.95^*^	**3.63 (2.44–5.4)**	279.96^*^	**5.41 (4.34–6.75)**
Renal tuberculosis^d^	(75)	*n.a*.	*n.a*.	*n.a*.	*n.a*.
Isoniazide^d^	(75)	n.r.	n.r.	n.r.	n.r.
Rifampin^d^	(75)	n.r.	n.r.	n.r.	n.r.
Ethambutol^d^	(75)	0.56	5.51 (0.78–39.21)	0.02	2.47 (0.35–17.58)
Pyrazinamide^d^	(75)	0.00016	2.04 (0.29–14.48)	1.81	2.75 (0.88–8.52)
Gestodene + Ethinylestradiol	(76)	n.r.	n.r.	n.r.	n.r.
Hepatitis B Virus	(77, 78)	*n.a*.	*n.a*.	*n.a*.	*n.a*.

a Disproportionality analyses were performed using OpenVigil (URL: http://openvigil.pharmacology.uni-kiel.de/openvigilfda.php). A possible association requires a minimum report count of N > 3, a reporting odds ratio > 2 and a Chi-Squared value > 4 (79). An asterisk (^*^) indicates a possible relationship between drug and disease. Drugs associated with both lichen planus and pemphigoid are indicated by bold font face.

b *n.r., respective condition not reported; n.a., no data available*.

c *Each of the references describes a single case of drug-induced LPP*.

d *The case report by Demirçay et al. (75) describes one patient with renal tuberculosis who developed LPP after treatment with isoniaziade, rifampin, ethambutol and pyrazinamide*.

### LPP and Infections

LPP has also been reported to be a complication of infection, particularly viral infections (Table 1), for example varicella (72, 73) and hepatitis B (77, 78). In fact, all of the published cases of LPP and viral hepatitis to date have been in association with hepatitis B (77, 78) and not hepatitis C infection. This is in contrast to LP, in which only hepatitis C has been reported as a trigger factor. The odds ratio to develop LP, especially oral LP, is described to be 2.5–4.5 for individual who are seropositive for HCV (80). Vice versa, a meta-analysis showed that the odds ratio to have a positive serology of HCV was 2.73–13.48 in patients with oral LP (81).

### LPP and Cancer

An association of LPP with colon adenocarcinoma points raises the possibility of a paraneoplastic variant of the disease (82).

### Medication as a Trigger of Both LP and BP

If one considers the possibility that LPP occurs as a complication of LP, it is conceivable that drugs that trigger the more prevalent disease LP also increase the risk of developing LPP. In fact, a large number of drugs have been described as possibly related to the development of LP, including ACE inhibitors. Several drugs are reportedly associated with the development of LP, including antimalarials, thiazide diuretics, NSAIDs, quinidine, beta-blockers, gold compounds, and tumor necrosis factor alpha inhibitors (51). The list of drugs that have been reported to induce BP is more extensive than the equivalent list for LP. It includes antibiotics, Calcium channel antagonists, ACE inhibitors, beta-blockers, angiotensin 1 antagonists, vaccines, NSAIDs, diuretics, Gliptins, TNF-alpha inhibitors, D-penicillamine, and tiobutarid (52). An induction of BP by PUVA has been reported only anecdotally (83, 84).

### Comparison of Case Report Data With Pharmacovigilance Information

Pharmacovigilance databases provide another valuable resource to identify drugs that may trigger the development of LPP. However, one major drawback is that LPP is not specified as a disease in the WHO list of adverse events. Instead, the terms “lichen planus” and “pemphigoid” were used to analyze pharmacovigilance data using the OpenVigil database tool (URL: http://openvigil.pharmacology.uni-kiel.de/openvigilfda.php). Pharmacovigilance report analyses are compared with case reports in Table 1, supported the observation of LPP cases that were caused by ACE inhibitors (enalapril, capropril, and ramipril), but also simvastatin and pembrolizumab. With the exception of captopril, both LP and pemphigoid disease were found to be putative side effects of these drugs. The reported association of anti-tuberculosis drugs with LPP could not be confirmed by pharmacovigilance data. For all other drugs and conditions that were suspected to be associated with LPP, no data could be found.

### Identification of Putative Triggers for LPP, LP, and BP From Pharmacovigilance Information

In addition, pharmacovigilance data also allows the identification of drugs that are not yet reported as possible triggers for LPP but are putative triggers for LP and/or pemphigoid (Table 2). Drugs that were reported as possible triggers for both LP and pemphigoid were hydrochlorothiazide, candesartan, sitagliptine, amlodipine, losartan, fluoxetine, and terbinafine. In addition, vildagliptine has been identified as a potent trigger for the development of BP (90), though OpenVigil did not contain any entries with this drug.

**Table 2:** Drugs and conditions not yet reported to be associated with LPP, but with LP or pemphigoid.

**Drug**	**Reference**^c^		**Pharmacovigilance data** **disproportionality analysis**^a,^ ^b^			
	**LP**	**Pemphigoid**	**Lichen planus**		**Pemphigoid**	
			**Chi squared (Yates)**	**Reporting odds ratio (95%-CI)**	**Chi squared (yates)**	**Reporting odds ratio (95%-CI)**
Ibuprofen	(51)	(52)	0.22	1.16 (0.72–1,87)	8.3^*^	**2.2 (1.3–3.8)**
**Hydrochlorothiazide**	(51)	(52)	190.2^*^	**4.9 (3.8–6.3)**	100^*^	**2.84 (2.3–3.5)**
Infliximab	(51)	(52)	36.9^*^	**2.9 (2.0–4.0)**	2.6	1.34 (0.9–1.9)
Etanercept	(51)	(52)	0.8	1.14 (0.9–1.5)	53.3^**^	**0.29 (0.2–0.4)**
Adalimumab	(51)	(52)	11.7	1.57 (1.2–2.0)	14.4^**^	**0.59 (0.5–0.8)**
Hydroxychloroquine	(51)		89.23^*^	**6.22 (4.1–9.5)**	2.6	0.37 (0.1–1.2)
**Candesartan**^**d**^	*no ref.^*e*^*		16.43^*^	**3.64 (2–6.8)**	149^*^	**6.09 (4.4–8.4)**
**Sitagliptine**	(85)	(52)	7.48^*^	**2.22 (1.29–3.9)**	1689^*^	**13.25 (11.3–15.6)**
Furosemide		(52)	0.56	1.2 (0.8–1.7)	382^*^	**3.96 (3.4–4.6)**
Verapamil		(52)	n.r.	n.r.	9.8^*^	**4.38 (1.82–10.5)**
Ciprofloxacine		(52)	0.04	1.2 (0.5–2.7)	13.4^*^	**2.14 (1.43–3.2)**
Amoxicillin	*no ref.^*e*^*	(52)	9.6^*^	**2.5 (1.4–4.5)**	0.07	1.13 (0.64–2)
Cephalexin		(52)	2.6	2.7 (1–7.1)	51.5^*^	**5.1 (3.2–8.2)**
Spironolactone		(52)	1.7	1.7 (0.9–3.4)	**13.9**	**2.2 (1.5**–**3.3)**
**Amlodipin**	(86)	(52)	33.3^*^	**2.3 (1.7**–**3.0)**	194^*^	**3.1 (2.6**–**3.7)**
**Losartan**	*no ref.^*e*^*	(52)	37.7^*^	**3.0 (2.1**–**4.4)**	**13**	**1.8 (1.3**–**2.5)**
Sulfasalazine	(87)	(52)	8.8^*^	**3.0 (1.5**–**6.0)**	0.03	0.8 (0.4–2)
**Fluoxetine**	*no ref.^*e*^*	(52)	19.4^*^	**2.6 (1.7**–**4.1)**	**15.4**	**2 (1.4**–**2.8)**
Gabapentine		(52)	0.1	0.9 (0.6–1.5)	17.8^**^	**0.3 (0.2**–**0.6)**
Galantamine hydrobromide		(52)	n.r.	n.r.	21.9^*^	**9.3 (3.5**–**24.9)**
Levetiracetam		(52)	0.07	1.2 (0.6–2.5)	23.3^*^	**2.4 (1.7**–**3.4)**
**Terbinafine**	(88)	(52)	8.4^*^	**4.8 (1.8**–**12.8)**	11.5^*^	**3.8(1.8**–**7.9)**
Omeprazole		(52)	11.9	1.7 (1.3–2.3)	9.7	1.4 (1.1–1.8)
Risperidone	(89)	(52)	0.8	0.5 (0.1–1.8)	19.6^*^	**2.5 (1.7**–**3.7)**
Hepatitis C virus	(51)		n.a.	n.a.	n.a.	n.a.

a Disproportionality analyses were performed using OpenVigil (URL: http://openvigil.pharmacology.uni-kiel.de/openvigilfda.php). A possible association requires a minimum report count of N > 3, a reporting odds ratio > 2 and a Chi-Squared value > 4 (79). An asterisk (^*^) and bold face indicates a possible relationship between drug and disease. Drugs associated with both lichen planus and pemphigoid are indicated by bold font face. A double asterisk (^**^) indicates a negative relationship between drug and disease. The corresponding drug names and relationship parameter values are highlighted in red.

b *n.r., respective condition not reported; n.a., no data available*.

c *Drug associations for pemphigoid as summarized by Stavropoulos et al. (52), for lichen planus as summarized by Arnold et al. (51) and reported by Swale and McGregor (86)*.

d *Candesartan was not reported by name previously, only by drug class*.

e *No ref. indicates that pharmacovigilance data analysis suggests an association of drug and disease, but the search term “lichen planus” plus drug name did not retrieve any pubmed records*.

For some drugs, pharmacovigilance report analyses do not overlap or even contradict the published literature (Table 2). In case of Ibuprofen, the association with pemphigoid could be confirmed, but not in case of LP. The same holds true for sulfasalazine and risperidone. In case of gabapentin, a negative association with pemphigoid was found. For TNFα inhibitors, pharmacovigilance data indicates a negative association of etanercept and adalimumab with pemphigoid and no association with LP. A negative association (odds ratio below 1) may indicate here a protective effect of the respective drug regarding pemphigoid, although gabapentin, etanercept and adalimumab are no established treatment options for this disease. Alternatively, this negative association may be the result of a statistical phenomenon where patients who tend to develop pemphigoid are less likely to be treated with the aforementioned drugs. In the other hand, infliximab seems to be positively associated with LP, but not with pemphigoid.

It has to be noted that pharmacovigilance data is based on physician-initiated reports about possible adverse events and not on a prospective data collection. Common belief about possible relationships between drugs and adverse events may lead to further skewing of data. Moreover, it is difficult to control for confounders or interactions. If a certain drug is used to treat a disease that predisposes for LP, BP or LPP, the disproportionality analysis cannot distinguish this from a drug-related predisposition. Furthermore, it is not possible to distinguish whether a certain drug truly causes LPP or whether it just increases the probability for a clinical manifestation in patients that are otherwise predisposed to LPP.

## Treatment

In a selective PubMed literature review of case reports published from the year 2000 onward, we were able to find treatment information from *N* = 53 patients in *N* = 43 articles (Table 3). Most reports (*N* = 42) describe the use of corticosteroids (mainly oral prednisolone) in various doses, from very low dose in some Japanese cases up to 2 mg/kg body weight. High doses of prednisolone are used for treatment of LPP in children. Topical corticosteroids were used in *N* = 20 cases, followed by dapsone in *N* = 15 cases.

**Table 3:** Reported treatment options for LPP since 2000.

**Treatment^a^**	**Success *N* cases**	Failure *N* cases	**References**
Acitretin	2	1	(70, 91, 92)
Azathioprin	3	1	(2, 17, 93, 94)
Ciclosporin (systemic)	2		(95, 96)
**Corticosteroids (systemic)**	**38**	**4**	(2, 5, 8, 12, 17, 26–28, 48, 62, 65, 67, 70–73, 75–77, 82, 91, 93–104)
**Corticosteroids (topically)**	**15**	**5**	(2, 8, 48, 49, 64, 72, 92, 93, 95, 97–99, 104–107)
Ciclosporine (topically)	Ø	1	(49)
**Dapsone**	**13**	**2**	(12, 26, 65, 66, 70, 75, 77, 91, 94, 98–100, 103, 108)
Doxycycline	1	Ø	(107)
Hydroxychloroquine	Ø	1	(94)
IVIG	3	Ø	(49, 62)
MTX	1	1	(95, 102)
Mycophenolatmofetile	2	1	(48, 62, 94)
PUVA	Ø	2	(70, 91)
Rituximab	Ø	1	(70)
Sirolimus	Ø	1	(70)
Tacrolimus (topically)	1	Ø	(48)
Tetracycline/Nicotinamide	2	1	(70, 94, 105)
Ustekinumab	1	Ø	(94)
**Termination of the triggering medication or tumor resection**	**8**	**2**	(49, 64–67, 69, 70, 76, 82, 93, 109)
Non-termination of potentially triggering medication	1	Ø	(70)

a *The listed treatment may be part of a combination therapy. Individual cases may be counted multiple times with different drugs. Treatment options with at least 10 reports are highlighted in bold font. Some publications report more than one case*.

In a case of oral lichen planus pemphigoides, topical gel ointment containing fluocinonid or dexamethasone was reportedly effective (48).

Although in most of the reported cases, patients were successfully treated with systemic corticosteroids, this might not be the best option given the side-effect profile. Other treatment options include topical corticosteroids, dapsone, and acitretin, whose use may be associated with fewer side-effects. This situation may be compared with that in BP. Here, systemic oral corticosteroids had been considered as standard treatment for decades (110) until Joly et al. effected a paradigmatic shift in treatment by demonstrating the equivalent efficacy of highly potent topical steroids, with fewer side-effects (111). On the other hand, given that patients with LPP tend to be younger, with fewer co-morbidities, one could argue that systemic treatment would be less hazardous and may result in speedier disease resolution.

Another interesting observation made during the review of the case reports is the very low doses of corticosteroids that were used for treatment in some Japanese cases (71, 97). Here, a dose of 15 mg, irrespective of body weight, was reportedly effective. In contrast, corticosteroid doses in cases from other countries are often between 0.5 and 1 mg/kg body weight, i.e., 2 to 4-fold higher.

From our experience (112), a combination of topical dexamethasone, prednisolone pulse therapy (100 mg/day for 3 days, initially every 3rd week) and acitretin (20 mg/day p.o.) is often sufficient to induce remission of blistering within 3 months and disappearance of LP lesions within 1 year. Similar observations have been reported in the literature (91).

Alternatives or additives to corticosteroid treatment could include dapsone (100 mg/daily), tetracycline (2 × 500 mg/daily) in combination with nicotinamide (2 × 500 mg/daily), mycophenolate mofetil (1000–1500 mg/daily in two doses), cyclosporine A (2 mg/kg body weight in 2 doses/day), methotrexate (7.5–20 mg/week or 0.5 mg/kg body weight/week in children).

Our PubMed literature search also retrieved the use of hypnotic suggestion for the treatment of LPP (113), though this article was published in 1959.

In 10 cases, a triggering medication or condition such as a underlying malignancy was identified and treated, resulting in resolution of skin lesions in 8 cases. One report describes the management of LPP associated with pembrolizumab therapy in a patient with malignant melanoma (70). In this patient, dapsone led to resolution of LPP, allowing the treatment with pembrolizumab to be continued. In this context, pembrolizumab was not necessarily the direct trigger of LPP *per se*, but may have been an important co-factor for the clinical manifestation of a subclinical disease, potentially related to its mode of action.

## Conclusion

LPP is a very rare disease entity that belongs to a larger family of diseases characterized by autoantibodies against COL17. It is a heterogenic disease, but shared common clinical features with other autoimmune blistering diseases, including BP and MMP. Its pathognomonic feature is the association with LP, supported by the demonstration of IgG and complement factor C3 deposition at the dermal-epidermal junction. Several case reports indicate an association with ACE inhibitors, simvastatin and checkpoint inhibitors, but also with HBV infection. There is no current consensus on the optimal treatment regime for LPP. However, combinations of systemic with topical corticosteroids, potentially in combination with dapsone or acitretin are worth considering. An improved understanding of the pathophysiology of LPP may help to shed new light on the mechanisms that lead to the development of autoantibodies against COL17 and subsequent blister formation.

## Author Contributions

FH, EL and AR discussed data and wrote the manuscript. AR identified the patient, performed literature search, and pharmacovigilance data analyses.

### Conflict of Interest Statement

The authors declare that the research was conducted in the absence of any commercial or financial relationships that could be construed as a potential conflict of interest.

## Footnotes

^1^Using multinomial regression (R open source statistical software, packages *nnet* for the function *multinom* and *car* for the function *Anova*) and the Benjamini-Hochberg correction for multiple testing.
